# The physiology of venoarterial extracorporeal membrane oxygenation - A comprehensive clinical perspective

**DOI:** 10.1177/02676591241237639

**Published:** 2024-04-23

**Authors:** Libera Fresiello, Jeannine A.J. Hermens, Lara Pladet, Christiaan L. Meuwese, Dirk W. Donker

**Affiliations:** 1 Cardiovascular and Respiratory Physiology, TechMed Center, University of Twente, Enschede, The Netherlands; 2 Intensive Care Center, 8124 University Medical Center Utrecht, Utrecht, The Netherlands; 3 Department of Cardiology, Thorax Center, 6993 Erasmus Medical Center, Rotterdam, The Netherlands; 4 Department of Intensive Care Adults, 6993 Erasmus Medical Center, Rotterdam, The Netherlands

**Keywords:** venoarterial extracorporeal membrane oxygenation, extracorporeal life support, temporary mechanical circulatory support, cardiovascular, ventriculo-arterial coupling

## Abstract

Venoarterial extracorporeal membrane oxygenation (VA ECMO) has become a standard of care for severe cardiogenic shock, refractory cardiac arrest and related impending multiorgan failure. The widespread clinical use of this complex temporary circulatory support modality is still contrasted by a lack of formal scientific evidence in the current literature. This might at least in part be attributable to VA ECMO related complications, which may significantly impact on clinical outcome. In order to limit adverse effects of VA ECMO as much as possible an indepth understanding of the complex physiology during extracorporeally supported cardiogenic shock states is critically important. This review covers all relevant physiological aspects of VA ECMO interacting with the human body in detail. This, to provide a solid basis for health care professionals involved in the daily management of patients supported with VA ECMO and suffering from cardiogenic shock or cardiac arrest and impending multiorgan failure for the best possible care.

## Introduction

Venoarterial extracorporeal membrane oxygenation (VA ECMO) is a widely used temporary circulatory support modality for severe cardiogenic shock and refractory cardiac arrest. In recent years, a surge of VA ECMO use in acute and critical cardiovascular care has been observed. Yet, this widespread daily practice still lacks robust scientific evidence to demonstrate convincing superiority of VA ECMO over conventional care.^1–4^ In part, this lack of evidence may be attributed to the complexity of VA ECMO patient management and related high rates of complications that significantly impact clinical outcomes. To minimize adverse effects and VA ECMO related complications, a proper understanding of the intricate patient-device interaction is pivotal. This, in turn, requires daily care based on dedicated qualification through education and training allowing health care professionals to anticipate and recognize unwanted effects of VA ECMO and timely adapt bedside management accordingly.

In more detail, VA ECMO provides arterialized blood flow to the systemic circulation in acute and severe cardiac, circulatory and often related respiratory failure. This support aims at stabilizing the patient’s shock state and reestablishing adequate multiorgan perfusion, while the arterialized extracorporeal blood flow into the aorta strongly influences cardiac and vascular function and conditions. This impact of VA ECMO on patient hemodynamics depends on the mode of cannulation, the configuration of the circuit and on how much extracorporeal support is provided. Also, individual patient characteristics and concomitant critical care support influence the interplay between device and human physiology dynamically.

Here, we set out to review and reflect on basic physiological concepts in VA ECMO to support bedside reasoning on individual patients and foster well-educated clinical management.

## Basic hemodynamic alterations explained

VA ECMO, often referred to as a miniaturized version of cardiopulmonary bypass (CPB), features a closed-loop, extracorporeal blood circuit which is much in contrast to its precursor, the traditional CPB, being an open system with a blood reservoir. In hemodynamic terms, this technical peculiarity of VA ECMO implies that the blood drained from the central venous circulation is directly reinfused into the aorta and its branches. The amount of venous drainage depends on the pressure drop across the VA ECMO circuit and the revolutions per minute set on the centrifugal blood pump, and tends to reduce central venous pressure, right atrial pressure and also right ventricular preload. The resultant effect is that VA ECMO may directly reduce right ventricular output and pulmonary circulation. The magnitude of this hemodynamic change also depends on patient factors such as blood volume status and compensatory baroreflex control. The oxygenated blood reinfused into the arterial circulation pressurizes the aorta, which may increase left ventricular afterload and indirectly also left ventricular preload, left atrial pressure and pulmonary pressure. Notably, the significance and dynamicity of this patient-device interaction is dependent on many different factors, e.g., the cause of cardiogenic shock, right-left ventricular interdependence, vascular tone, volume status, etc. Taken together, the native and the extracorporeal blood circuit interact in parallel as two quasi closed loop circuits. From this perspective, the blood infused via the extracorporeal circuit is eventually reentering the central venous blood pool. This also means that the infusion of extracorporeal blood flow into the arterial compartment does not directly dictate left ventricular afterload but should be considered as affecting a dynamic hemodynamic equilibrium between arterial and venous circulation. Herein, also the native and artificial blood pumps, i.e., the right ventricule, the left ventricule, the atria and the extracorporeal centrifugal pump act interdependently and are interconnected with the different vascular beds being the central venous, pulmonary and systemic arterial circulation, respectively. At the bedside, all these aspects should be taken into account when assessing the patient’s circulatory status under VA ECMO support.

## Cardiac function and loading

A frequent and thorough assessment of the patient’s cardiac function and geometry is of pivotal importance during VA ECMO support as cardiac recovery and weaning from the extracorporeal circulation are key objectives during clinical care.

Transthoracic and transesophageal echocardiography allow to collect detailed anatomical and functional information on the heart’s condition.^5^ During VA ECMO, echocardiographic investigation should therefore be performed by experienced operators as part of the daily routine and whenever the clinical situation requires it.

### Cardiac mechanical loading conditions

In addition to the clinical aim of hemodynamic and respiratory stabilization of the patient’s cardiogenic shock state, it is also important to optimally manage VA ECMO with the objective to avoid cardiac overloading and promote myocardial recovery. It has been described clinically and experimentally that VA ECMO can increase left ventricular mechanical loading, i.e., preload and afterload.^6–14^ In this context, it is often suggested that the retrograde infusion of VA ECMO-derived blood into the aorta importantly dictates left ventricular overload. This phenomenon is encountered in peripherally cannulated VA ECMO which is the most commonly used technique. Yet, also central VA ECMO pressurizes the aorta through non-pulsatile, continuous infusion of oxygenated blood and may thus also increase left ventricular afterload. So far, it remains to be elucidated how the degree of extracorporeal support flow, specific cannula positions and related aortic blood flow dynamics as well as patient factors impact left ventricular afterload. Experimental and clinical data remain ambiguous underlining the complexity of this matter.^9,15^

For daily clinical care, it should be emphasized that arterial blood pressure control, e.g., striving for a mean value of 65 mmHg and reducing hypervolemia as much as possible can significantly contain left ventricular afterload.^12^ Also, reducing the extracorporeal blood flow to a tolerable minimum required for an adequate systemic circulation can reduce left ventricular afterload and prevent consequential LV dilatation.^5,12^

As part of the daily routine, cardiac function and loading conditions under VA ECMO should be assessed through advanced continuous hemodynamic monitoring, preferentially with a pulmonary artery catheter combined with repeated bedside echocardiography. In this context, it should be underscored that assessment of right ventricular function is pivotal, as a right ventricle with preserved contractility effectively pumps blood into the left side of the heart, predisposing for left ventricular overload during VA ECMO.^16^

When conservative management of cardiac loading conditions during VA ECMO is insufficient, adjunct left ventricular unloading interventions can be considered. These additional therapies should be evaluated carefully in terms of benefits and related complications (e.g. bleeding and limb ischemia).^17,18^ Also, patient-specific clinical goals need to be clearly defined:

• Unloading or decompressing refers to strategies aimed at reducing the mechanical (over-)load imposed on the left ventricle that in first approximation depends on cavity pressures and geometry (Laplace’s law)
• Venting strategies are aimed to assure transpulmonary and transcardiac blood flow to prevent thrombosis in the left ventricular cavity and aortic root
• Combined mechanical circulatory support strategies using VA ECMO in conjunction with another device may allow to increase the total systemic blood flow beyond the limits of VA ECMO alone

These therapeutic objectives of adjunct interventional left ventricular unloading or venting strategies in VA ECMO may clinically be intertwined but represent distinct physiological facets of combined mechanical circulatory support.

### Assessment and management of cardiac function

A proper characterization of the myocardial contractility of the failing or recovering heart during VA ECMO can be challenging due to the complex interaction of the patient’s cardiovascular system with the extracorporeal blood circuit. During VA ECMO, the right ventricle might be significantly unloaded while the left ventricle might experience an increased afterload. This in turn may impact on right-left ventricular interdependence influencing pulmonary blood flow and left ventricular loading.^16^ These aspects become particularly relevant during the process of weaning from VA ECMO. Various hemodynamic and echocardiographic parameters have been investigated to assess patients’ readiness and the expected success for weaning. Although most of these parameters focus on the left heart status,^19^ it should be underscored that right ventricular function is also vital for long-term outcome, especially when aiming for chronic mechanical circulatory support.

**Figure 1.**
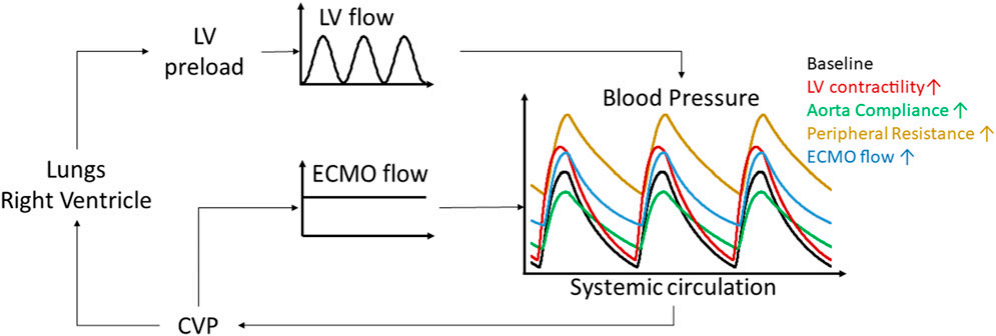
Schematic representation of the human cardiovascular system interacting with the venoarterial extracorporeal membran oxygenation (VA ECMO) circuit. The pulmonary circulation is represented by the elements central venous pressure (CVP), right ventricle and lungs, connecting to the systemic circulation represented by left ventricular (LV) preload, resulting in a pulsative native LV flow and the systemic blood pressure through ventriculo-arterial coupling. The VA ECMO circuit drains central venous blood (CVP), generates a continuous extracorporeal blood flow (ECMO flow) infused into the systemic circulation and contributes to the systemic blood pressure through ECMO-arterial coupling. As a result of ventriculo-arterial and ECMO-arterial coupling a pulsatile arterial waveform can be observed, its pulsatility, magnitude and shape being influenced by increased LV contractility (red), increased aortic compliance (green), increased peripheral resistance (ochre) and increased ECMO blood flow.

Alternatively, the heart’s contractile function under VA ECMO can also be monitored indirectly at the bedside by assessing the peripheral arterial blood pressure tracings, i.e., the arterial pulse pressure defined as the difference between the systolic and diastolic blood pressure.^20^ Clinically, it is well appreciated that arterial pulsatility decreases when increasing VA ECMO blood flow, while mean blood pressure usually rises due to the aortic pressurization through continuously infused extracorporeal blood flow.^21^ Arterial pulse pressure seems to hold important prognostic information in VA ECMO in cardiogenic shock and cardiac arrest.^22–27^ In order to further clarify its role as a surrogate measure of cardiac contractility more research is needed in order to elucidate its intricate nature under VA ECMO. From a hemodynamic point of view, arterial pulse pressure is the result of left ventricular pulsatile flow combined to the VA ECMO flow infused into the aorta or one of its large branches. The ECMO flow is continuous and non-pulsatile compared to the native ventricular flow, being mainly the result of changes in pressure drop across the circuit and centrifugal blood pump characteristics. When interpreting arterial pulse pressure, waveform and mean pressure, cardiac function and arterial blood flow aspects should be considered together with characteristics related to the arterial vasculature, e.g., the aortic compliance being decreased with age and vascular disease, and the peripheral vascular resistance modified by patient factors or vasoactive medication (Figure 1). Obviously, also heart rate, intravascular blood volume and inotropic and vasopressive support may all influence the patient’s contractile reserve and mask an objective assessment of the heart’s native contractility.^28^

As a clinical reference, a minimal pulse pressure of 10 mmHg is proposed in international guidelines,^29^ in order to assure transpulmonary and cardiac flow, left ventricular ejection and prevent serious complications as ventricular cavity and aortic root thrombosis. A specific arterial pulse pressure does not equal (in)adequate cardiac mechanical loading conditions, but a low arterial pulse pressure, i.e., <10 mmHg warrants a detailed analysis of cardiac function and loading conditions.

Also, when assessing weaning readiness, arterial pulse pressure can be considered, while it should be noted that a value <15 mmHg has been associated with low cardiac output.^23^ In general, weaning from VA ECMO requires a systematic, meticulous assessment of the patient’s hemodynamics in relation to the degree of VA ECMO support and adjuvant critical care, and especially when standard approaches to weaning are not successful.^19,30^ Such detailed clinical assessment should also include the differential diagnosis of cardiac tamponade, particularly in cases when weaning is difficult or poorly understood as classical clinical signs of tamponade may not be present during VA ECMO due to the influences of patient-device interaction on hemodynamics.^30,31^

### Optimal monitoring and management of cardio-pulmonary function and condition

When high extracorporeal blood flow rates (>4 L/min; 60-80 mL/kg) are required to maintain adequate organ perfusion,^32^ it should be realized that pulmonary artery blood flow may significantly decrease.^33^ This may potentially cause ischemia of the native lung.^34,35^ Although, the lung has a dual blood supply, the perfusion of the pulmonary parenchyma importantly depends on pulmonary arterial flow and cannot fully be compensated through bronchial arterial blood supply. Especially, patients supported with VA ECMO after lung transplant have an additional risk for pulmonary and tracheobronchial ischemia, as during transplantation the bronchial arteries are routinely sacrificed. As an indication, a critical threshold might be reached when reducing pulmonary artery flow to <25% of the total cardiac output for a prolonged period of time.^34,35^ Clinically, it is crucial to be aware of this underlying pathophysiology resulting from high flow VA ECMO as pulmonary ischemia can remain obscure and unrecognized at the bedside and it may only become evident through clinical signs of pulmonary edema. Importantly, pulmonary arterial blood flow should be monitored carefully using a pulmonary artery catheter, echocardiography, or indirectly through levels of end-tidal carbon dioxide (EtCO_2_) by capnography during mechanical ventilation.^23^

Monitoring the pulmonary circulation should always be performed in conjunction with the assessment of right ventricular function, while expecting a reduced right ventricular preload under VA ECMO. Yet, in this context, pulmonary vascular resistance (PVR) plays a key role as it augments both right ventricular afterload and preload.

An increased PVR can be the result of volume overload, pre-existing and long-standing left-sided heart failure, which may only in part be reversible. In addition, hypercarbia in the context of shock but also resulting from pulmonary abnormalities including edema, atelectasis and infiltrative changes may all increase PVR. Hypercarbia should be avoided by adjusting mechanical ventilatory settings or extracorporeal gas flow. In this way, aiming for normocarbia will prevent an increase in PVR thereby warranting sufficient pulmonary blood flow with acceptable right ventricular loading conditions.

### Pulmonary complications

As a consequence of left ventricular overload occuring during VA ECMO, high left ventricular filling pressure resulting from the underlying heart failure may further increase and cause or exacerbate pulmonary edema, a common complication of VA ECMO.^10,36^ This pulmonary edema may further be complicated by pulmonary ischemia resulting from high-flow VA ECMO bypassing the pulmonary circulation as well as systemic inflammation due to shock, concurrent infection or blood contact with artificial surfaces of the VA ECMO circuit, as recently reviewed.^37^

Pulmonary complications may significantly impair native gas exchange that results in poorly oxygenated blood ejected from the left heart into the aorta. This leads to the well-known phenomenon of differential hypoxemia or Harlequin’s syndrome critically compromising upper body oxygenation, particularly the coronary and cerebral circulation. Harlequin’s syndrome may mainly occur in peripheral VA ECMO when cardiac contractility recovers, but also in presence of additional blood pumps that supports ventricular ‘ejection', i.e., ECPELLA or ECMELLA combining VA ECMO and Impella^R^.^38,39^ At the bedside, Harlequin’s syndrome should immediately be counteracted by optimization of mechanical ventilatory settings, i.e., increase of positive end-expiratory pressure (PEEP) and fraction of inspired oxygen (FiO2) and correction of hypervolemia. When conservative measures fail, veno-venous arterial, semi-central subclavian or central VA ECMO configuration should be considered. Alternatively, a strictly selective upper body central venous drainage in the superior caval vein has been proposed to prevent differential hypoxemia.^40^

### Right and left ventriculo-arterial coupling and interdependence

**Figure 2.**
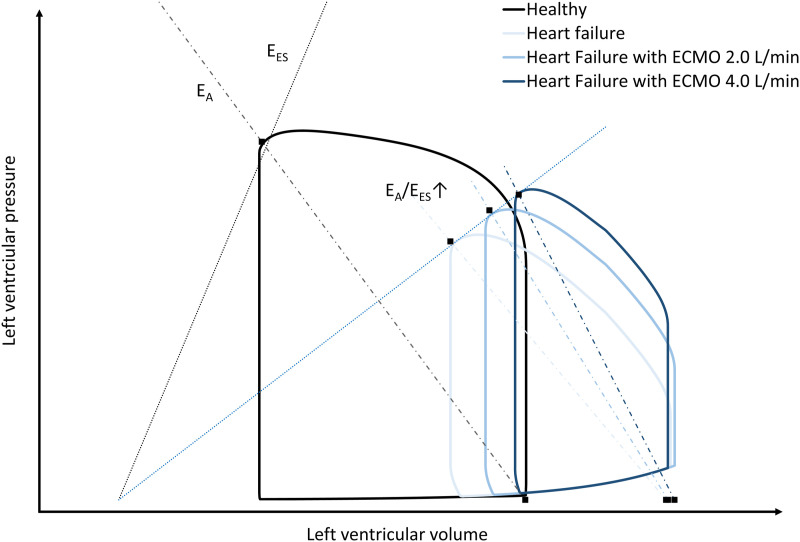
Schematic representation of pressure-volume loops of the left ventricle during baseline (Healthy), heart failure and heart failure supported with different blood flows provided by venoarterial extracorporeal membrane oxygenation (ECMO). E_A_ indicates arterial elastance, E_ES_ indicates ventricular elastance, see text for details.

The concept of ventriculo-arterial coupling refers to the heart and the arterial vascular tree as interdependent systems creating cardiac output and blood pressure.^41^ Ventriculo-arterial coupling can be expressed as the ratio of arterial elastance (E_A_) and ventricular elastance (E_ES_), the E_A_/E_ES_ ratio, with a normal reference around 1.0 mmHg/ml (Figure 2). Clinically, E_ES_ is a parameter related to the myocardial contractility and refers to the ratio between the left ventricular end-systolic pressure and volume, with a normal reference around 2.5 mmHg/ml.^42^ E_A_ represents the effective arterial elastance, a parameter representing the extracardiac forces or arterial load the ventricle has to overcome during ejection, with a normal reference around 2 mmHg/ml. Ventriculo-arterial coupling allows to estimate how efficiently the heart pumps blood into the arterial vasculature. In heart failure and during VA ECMO support, the E_A_/E_ES_ ratio can strongly be increased interpretable as relatively increased afterload or E_A_ compared to decreased ventricular contractility or E_ES_, commonly coined as ventriculo-arterial *un*coupling. In contrary, E_A_/E_ES_ ratios <0.5 mmHg/ml suggest optimal ventriculo-arterial coupling, the heart effectively contracting against a low vascular resistance. The concept of ventriculo-arterial coupling may be used to clinically assess the patient’s cardiovascular condition under VA ECMO and has been reported for different cardiovascular diseases, but deserves more experimental and clinical analyses.^42,43^ From a clinical perspective, it should be noted that bedside use of E_A_ and E_ES_ is not a widespread routine but can be conducted using echocardiography and invasive arterial blood pressure monitoring.^42^

In analogy to left-sided ventriculo-arterial coupling, right-sided ventriculo-pulmonary arterial coupling and right-left ventricular interaction or interdependence are also important physiological concepts that affect daily management of VA ECMO.^16^ Right ventriculo-pulmonary arterial coupling has also been described as proportional pulmonary pulse pressure, the ratio of pulmonary artery pulse pressure to mean pulmonary artery pressure. Proportional pulmonary pulse pressure has been reported to be decreased in patients with left ventricular distention during VA ECMO and increased in the absence of distention, reflecting left ventricular mechanical overload during VA ECMO.^44^ This notion emphasizes the importance of right ventricular contractility for left ventricular overload as based on right-left ventricular interdependence.^16^ Transthoracic echocardiography allows to assess right ventriculo-pulmonary arterial coupling during VA ECMO non-invasively through echocardiographic measures of right-ventricular contractility in relation to estimates of right ventricular systolic pressure.^45^

The amount of transpulmonary flow can also simply be monitored at the bedside based on the relation between transpulmonary blood flow and EtCO_2_ as measured in the exhaled gas during mechanical ventilation, both being intrinsically related to right ventricular function, pulmonary blood flow and right-left ventricular interdependence. In this context, a critical threshold indicative of a low cardiac output is an EtCO_2_ <14 mmHg,^23^ whereas a significant rise of EtCO_2_ >5 mmHg has been associated with successful weaning from VA ECMO.^46^

**Figure 3.**
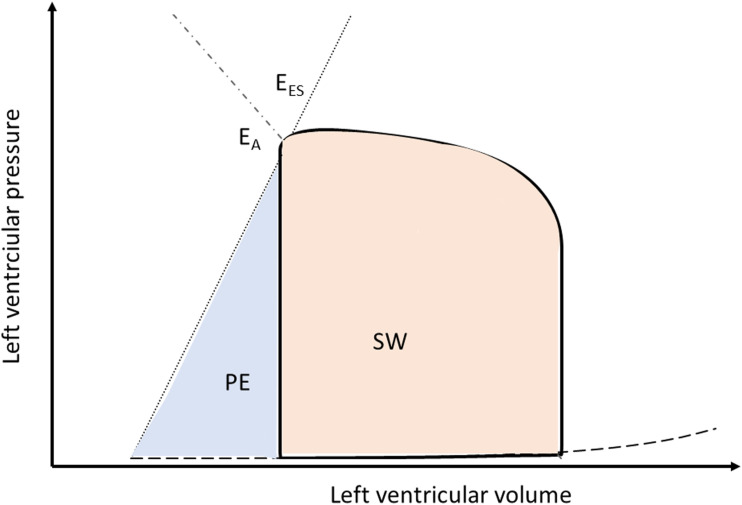
Schematic representation of a pressure-volume loop of the left ventricle illustrating stroke work (SW) and potential energy (PE). E_A_ indicates arterial elastance, E_ES_ indicates ventricular elastance, see text for details.

The analysis of ventriculo-arterial coupling also allows to estimate cardiac energetics based on the interaction of the heart with the vascular system. In the pressure volume loop stroke work (SW) and potential energy (PE) can be identified, its sum, the pressure volume area, equals the total energy consumed during a cardiac cycle which is related to myocardial oxygen consumption (Figure 3). Herein, SW represents the energy transmitted during ejection of the ventricle to maintain arterial blood pressure, while ventricular efficiency is expressed as the ratio of SW and the pressure volume area and PE the energy stored in the myocardium at end-systole (Figure 3).

## Conclusions

Close monitoring of cardiovascular function during VA ECMO is challenging due to the complex and dynamic interaction between the patient and the extracorporeal device.

An in-depth understanding of the physiological mechanisms and concepts helps to optimally tailor VA ECMO support to the patient’s needs, promote cardiac recovery and prevent complications.

Continuing education and training of health care professionals involved in daily ECMO care should importantly be based on knowledge of human pathophysiology supported by advanced hemodynamic assessment and aimed at well-educated bedside reasoning for the best possible patient care.
